# Characterization of Multi-Drug Resistant *Enterococcus faecalis *Isolated from Cephalic Recording Chambers in Research Macaques (*Macaca* spp.)

**DOI:** 10.1371/journal.pone.0169293

**Published:** 2017-01-12

**Authors:** Stephanie E. Woods, Mia T. Lieberman, Francois Lebreton, Elise Trowel, César de la Fuente-Núñez, Joanne Dzink-Fox, Michael S. Gilmore, James G. Fox

**Affiliations:** 1 Division of Comparative Medicine, Massachusetts Institute of Technology, Cambridge, Massachusetts, United States of America; 2 Department of Biological Engineering, Massachusetts Institute of Technology, Cambridge, Massachusetts, United States of America; 3 Departments of Ophthalmology, and Microbiology and Immunobiology, Harvard Medical School, Massachusetts Eye and Ear Infirmary, Boston Massachusetts, United States of America; 4 Synthetic Biology Group, MIT Synthetic Biology Center, Massachusetts Institute of Technology, Cambridge Massachusetts, United States of America; 5 Research Laboratory of Electronics, Massachusetts Institute of Technology, Cambridge, Massachusetts, United States of America; 6 Department of Electrical Engineering and Computer Science, Massachusetts Institute of Technology, Cambridge, Massachusetts, United States of America; 7 Broad Institute of MIT and Harvard, Cambridge, Massachusetts, United States of America; 8 Harvard Biophysics Program, Harvard University, Boston, Massachusetts, United States of America; 9 The Center for Microbiome Informatics and Therapeutics, Cambridge, Massachusetts, United States of America; University of Kansas, UNITED STATES

## Abstract

Nonhuman primates are commonly used for cognitive neuroscience research and often surgically implanted with cephalic recording chambers for electrophysiological recording. Aerobic bacterial cultures from 25 macaques identified 72 bacterial isolates, including 15 *Enterococcus faecalis* isolates. The *E*. *faecalis* isolates displayed multi-drug resistant phenotypes, with resistance to ciprofloxacin, enrofloxacin, trimethoprim-sulfamethoxazole, tetracycline, chloramphenicol, bacitracin, and erythromycin, as well as high-level aminoglycoside resistance. Multi-locus sequence typing showed that most belonged to two *E*. *faecalis* sequence types (ST): ST 4 and ST 55. The genomes of three representative isolates were sequenced to identify genes encoding antimicrobial resistances and other traits. Antimicrobial resistance genes identified included *aac(6’)-aph(2”)*, *aph(3’)-III*, *str*, *ant(6)-Ia*, *tetM*, *tetS*, *tetL*, *ermB*, *bcrABR*, *cat*, and *dfrG*, and polymorphisms in *parC* (S80I) and *gyrA* (S83I) were observed. These isolates also harbored virulence factors including the cytolysin toxin genes in ST 4 isolates, as well as multiple biofilm-associated genes (*esp*, *agg*, *ace*, *SrtA*, *gelE*, *ebpABC*), hyaluronidases (*hylA*, *hylB*), and other survival genes (*ElrA*, *tpx*). Crystal violet biofilm assays confirmed that ST 4 isolates produced more biofilm than ST 55 isolates. The abundance of antimicrobial resistance and virulence factor genes in the ST 4 isolates likely relates to the loss of CRISPR-*cas*. This macaque colony represents a unique model for studying *E*. *faecalis* infection associated with indwelling devices, and provides an opportunity to understand the basis of persistence of this pathogen in a healthcare setting.

## Introduction

**Fig 1:**
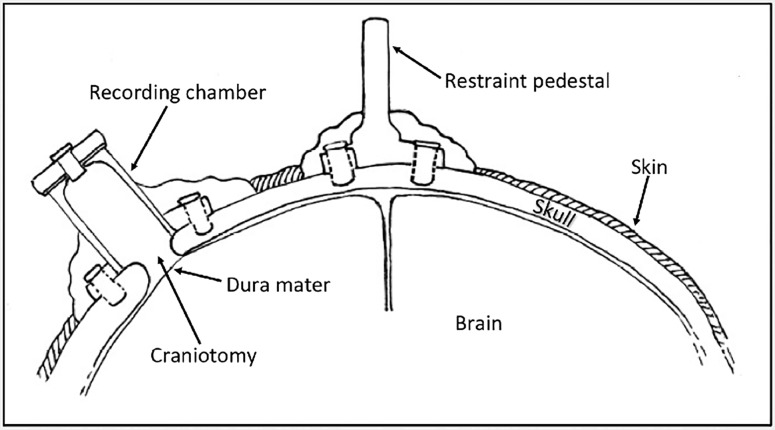
Illustration of Cephalic Implants Used for Neuroscience Research. Cephalic recording chambers and restraint pedestals are commonly affixed to the skull using a combination of screws and polymethyl methacrylate acrylic or other polymers.

Nonhuman primates (NHP) are an important animal model in cognitive neuroscience research, with the macaque (*Macaca spp*.*)* being the most commonly utilized species [1]. The anatomical and functional similarities between the human and macaque brain have been well characterized, and features such as a highly developed cerebral cortex, binocular color vision and front-facing eyes allow comparisons to humans that are impossible in rodent models [1]. Implanted cephalic chambers allow placement of microelectrodes into specific regions of the brain to monitor the activity of individual neurons (Fig 1). Materials commonly used in cephalic recording chamber implants include CILUX plastic (CRIST Instruments), titanium, stainless steel, thermoplastic polyetherimide Ultem (Gray Matter Research), polymethyl methacrylate acrylic and combinations of these materials. Due to the long-term nature of neurophysiology studies, surgically implanted macaques can be maintained for 10 years or longer. A major complication of chronic cephalic implantation is bacterial infection of the recording chambers, with occasional subclinical and clinical cases of meningitis and rare cases of cephalic abscessation [2–5]. Previous publications have reported *Staphylococcus aureus*, *Corynebacterium ulcerans*, *Candida* sp. and *Trichosporon beigelii* as common bacterial and fungal contaminants of recording chambers and *S*. *aureus*, *Streptococcus* spp., *Corynebacterium* spp., and *Enterococcus* spp. as common bacterial isolates from the skin-cranial implant margin [4–6]. Previously, *C*. *ulcerans* isolated from the skin-cranial implant margin was found to be sensitive to commonly used antimicrobials, however limited information is available on antimicrobial sensitivities of bacterial species isolated from within cephalic recording chambers. The first goal of this study was to identify microbes colonizing cephalic recording chambers in our macaque colony, and evaluate antimicrobial resistance profiles that may be important for intervention. We hypothesized that antimicrobial resistant microbes would be likely, due to intermittent topical and systemic antimicrobial administration. We identified multi-drug resistant *Enterococcus faecalis* as the second most prevalent species in recording chambers. *E*. *faecalis* is an important nosocomial pathogen of humans, and its ability to form biofilm contributes to catheter-associated blood and urinary tract infections [7, 8]. Recording chambers offer an interface for biofilm formation in cranially-implanted macaques, and we hypothesized that *E*. *faecalis* isolated from recording chambers would have similar sequence types, virulence factors, and biofilm genes as human isolates. We highlight the value of implanted macaques as a model of human *E*. *faecalis* infection associated with long term indwelling devices.

## Materials and Methods

### Animals

Twenty-five macaques (19 male, 6 female) with chronic cephalic recording chambers were sampled in August, 2011. The population consisted of 24 rhesus macaques (*Macaca mulatta*) and 1 cynomolgus macaque (*Macaca fasicularis)*, with a mean and standard deviation age of 10.7 ± 2.2 years (range 6–15 years). All animals were sourced from USDA-approved vendors. Select population characteristics are shown in Table 1. Animals were housed in accordance with *The Guide for Care and Use of Laboratory Animals* in an AAALAC International-accredited facility. All animal protocols were approved by the Committee on Animal Care at the Massachusetts Institute for Technology. Routine husbandry parameters at the institution include a 12:12 light/dark cycle, and provision of a commercially formulated primate diet (Purina 5038) supplemented with a foraging mixture of fruit, vegetables, nuts and cereal. Water is provided *ad libitum* except when water regulation is required under approved protocols. Environmental enrichment includes positive interaction with care staff, treats, food puzzles, toys, mirrors, and videos. Macaques are pair-housed, except when precluded by established behavioral incompatibility or for medical reasons, as determined by a veterinarian. The macaques sampled in this study were under investigation by four different cognitive neuroscience research laboratories (A-D; Table 1). For this study, 44 cephalic recording chambers with craniotomies, and 1 without a craniotomy, were sampled. The majority of macaques (11/25) had one cephalic recording chamber; 8/25 had two, and the remaining 6 macaques had three cephalic recording chambers (Table 1). Most (26) of the recording chambers had been in place between one and three years, 12 had been implanted between three and five years, and 7 had been in place less than one year. To minimize discomfort and stress, sampling of cephalic chambers was performed under ketamine anesthesia (10mg/kg, intramuscular injection) during routine semi-annual physical examination. All animals remained on IACUC-approved protocols for cognitive neuroscience at the conclusion of sampling and no animals were euthanized for reasons related to this study.

**Table 1:** Population Characteristics of Sampled Research Macaques.

Animal ID	Sex	Age (years)	Laboratory ID	# of Recording Chambers	*E*. *faecalis* Isolates
1	F	15	A	1	Isolate 1
2	F	14	A	1	
3	F	12	A	1	
4	M	11	B	2	Isolate 2
5	M	10	B	3	Isolates 3, 4, 5
6	M	13	B	2	Isolate 11
7	M	12	B	3	
8	M	13	B	3	Isolate 12
9	M	13	B	3	Isolate 13
10	M	10	B	1^1^	
11	M	10	B	2	Isolates 14, 15
12	M	12	C	2	
13	M	12	C	3	
14	M	11	C	2	
15	M	8	C	1	Isolate 6
16	F	10	C	2	Isolates 7, 8
17	F	11	C	3	
18^2^	M	10	C	2	Isolate 9
19	M	10	C	2	Isolate 10
20	F	11	D	1	
21	M	8	D	1	
22	M	7	D	1	
23	M	7	D	1	
24	M	11	D	1	
25	M	6	D	1	

^1^This macaque had a cephalic recording chamber without a craniotomy

^2^Cynomolgus macaque

### Recording Chamber Maintenance and Condition

Primary caregivers were surveyed for the frequency of cephalic recording chamber cleaning, gross appearance of recording chamber discharge, and usage of topical antimicrobial agents and packing material in the chamber (S1 Table). Overall, cephalic recording chambers were typically disinfected daily during periods of recording for most animals, and were cleaned between three times a week to every 3–4 weeks for those not actively being studied. Materials and antimicrobials used in the chambers by different investigative teams varied, and included sterile non-woven sponge balls (Super Stoppers, Metropacifica LLC, Nashville, TN), sterile petroleum jelly, gentamicin sulfate 0.3% ophthalmic solution (Gentak, Akorn Pharmaceuticals, Lake Forest, IL), bacitracin zinc 400U/g-neomycin sulfate 5mg/g-polymyxin B 10,000U/g ophthalmic ointment (Akorn Pharmaceuticals, Lake Forest, IL), 0.5% oxytetracyline with polymixin B (Terramycin, Zoetis, Florham Park, NJ) and a 1:20 dilution of injectable 22.7 mg/ml enrofloxacin (Bayer, Shawnee Mission, Kansas).

### Bacterial Culture

Sterile culture swabs (CultureSwab MaxV(+), BD, Franklin Lakes, NJ) were used to sample the interior of cephalic recording chambers, including any discharge present. The swabs were plated onto chocolate agar, trypticase soy agar with 5% sheep blood and MacConkey agar plates and incubated at 37°C in 5% CO_2_ for 24 h. The swabs, themselves, were then incubated in thioglycollate broth at 37°C for 24 h and re-plated onto the media listed above. Microbial growth was streaked onto blood agar to obtain isolated colonies, which were then identified using the Analytical Profile Index identification system (API 20 E and API 20 Strep, bioMérieux, Durham, NC).

### Antimicrobial Susceptibility Testing

Initial antibiotic susceptibility profiles were determined by disk diffusion using the Clinical and Laboratory Standards Institute break points (M2-A10) [9]. Antimicrobial agents tested included ampicillin, amoxicillin/clavulanic acid, bacitracin, cephalothin, erythromycin, gentamicin, oxacillin, trimethoprim-sulfamethoxazole, enrofloxacin, tetracycline, oxytetracycline, ceftriaxone, doxycycline, neomycin, cefazolin, polymyxin B and vancomycin. Chloramphenicol, gentamicin, streptomycin, and vancomycin disks were purchased from BD (BBL Sensi-Disc, Franklin Lakes, New Jersey); the remainder of the antimicrobial disks were obtained from Oxoid (Basingstoke, United Kingdom). Minimum inhibitory concentrations for key antibiotics were determined by broth microdilution in CAMHB (Cation-Adjusted Muëller Hinton Broth) as recommended [10]. For daptomycin MIC determination, the CAMHB was supplemented with calcium to a final concentration of 50 μg/ml. Amoxicillin-clavulanic acid and trimethoprim-sulfamethoxazole combinations were tested for MIC using Etest strips according to the manufacturer’s instructions (Etest, bioMérieux, Durham, NC). The MIC was read at the lowest concentration where the ellipse of inhibited growth intersected the testing strip. All antibiotics used for broth microdilution were purchased from Sigma-Aldrich Chemical Company (St Louis, MO). Control strains included vancomycin-susceptible ATCC *E*. *faecalis* 29212 and vancomycin-resistant ATCC *E*. *faecalis* 51299.

### DNA Extraction

DNA was extracted from overnight broth cultures of *E*. *faecalis* using a commercially available kit (Qiagen DNeasy Blood and Tissue Kit, USA). Manufacturer instructions were modified by the addition of 50 μl of lysozyme (50 mg/ml) and 10 μl mutanolysin (2500 U/ml, Sigma-Aldrich) during a 30 minute incubation at 37°C before the addition of proteinase K and buffer.

### PCR and MLST

Polymerase chain reaction (PCR) amplification of the D-alanine:D-alanine ligase gene (*ddl*), 16S rRNA gene, and MLST genes were performed using the listed primers (Table 2), with amplification conditions based on previously published protocols [11, 12]. PCR products were separated by electrophoresis through a 1% agarose gel at 100-120V for 30–40 minutes, prior to ethidium bromide staining and visualization with UV light. Prior to sequencing, PCR products were purified using a commercial kit according to manufacturer instructions (Qiagen, USA), or purified prior to sequencing by a commercial laboratory (QuintaraBio, Cambridge, MA). Purified PCR products underwent Sanger sequencing at the DNA Core Facility at the Massachusetts General Hospital Center for Computational and Integrative Biology, or at a commercial laboratory (QuintaraBio, Cambridge, MA). Sequence types were identified using the *Enterococcus faecalis* MLST website (http://pubmlst.org/efaecalis) at the University of Oxford [13].

**Table 2:** Primers Used for the Identification and Characterization of *E*. *faecalis*.

Primer Set	Sequence (5'-3')	Amplicon Size (bp)	Reference
*E*. *faecalis ddl* gene	CAGAAGTGAAGAGCACGATG	647	In-house design
	AGGTAAAGTCGTACGGACAT		
16S universal primer	AGAGTTTGATCCTGGCTGAG	1550	Coenye, T., et al.^11^
	AAGGAGGTGATCCAGCCGCA		
*pstS*—MLST	CGGAACAGGACTTTCGC	583	Ruiz-Garbajosa, P., et al.^12^
	ATTTACATCACGTTCTACTTGC		
*aroE*—MLST	TGGAAAACTTTACGGAGACAGC	459	Ruiz-Garbajosa, P., et al.^12^
	GTCCTGTCCATTGTTCAAAAGC		
*gdh*—MLST	GGCGCACTAAAAGATATGGT	530	Ruiz-Garbajosa, P., et al.^12^
	CCAAGATTGGGCAACTTCGTCCCA		
*gyd*—MLST	CAAACTGCTTAGCTCCAATGGC	395	Ruiz-Garbajosa, P., et al.^12^
	CATTTCGTTGTCATACCAAGC		
*gki*—MLST	GATTTTGTGGGAATTGGTATGG	438	Ruiz-Garbajosa, P., et al.^12^
	ACCATTAAAGCAAAATGATCGC		
*xpt*—MLST	AAAATGATGGCCGTGTATTAGG	456	Ruiz-Garbajosa, P., et al.^12^
	AACGTCACCGTTCCTTCACTTA		
*yiqL*—MLST	CAGCTTAAGTCAAGTAAGTGCCG	436	Ruiz-Garbajosa, P., et al.^12^
	GAATATCCCTTCTGCTTGTGCT		

### Whole Genome Sequencing, Antimicrobial Resistance Genes and Virulence Factor Identification

*E*. *faecalis* isolates #1, #12, and #13, from macaques 1, 7, and 8, respectively, were each sequenced on a single SMRT cell on a Pacific Biosciences RS2 at the University of Massachusetts Deep Sequencing Core Facility. DNA libraries were prepared for sequencing with the SMRTbell Template Prep Kit 1.0 and the DNA/Polymerase Binding Kit P6 v2, according to manufacturer instructions (Pacific Biosciences, Menlo Park, CA). A total of 87,196, 93,299 and 85,195 reads were obtained for genomes from isolates 1, 12 and 13, respectively; resulting in 3 polished contigs for isolates 1 and 12, and 2 polished contigs for isolate 13. N50 read lengths were 24,838, 24,224 and 23,305 bases, and average reference coverage was 327.48, 338.83 and 293.2 for isolates 1, 12, and 13, respectively. Filtered subreads were assembled *de novo* using the Hierarchical Genome Assembly Process (HGAP 3.0) workflow, with the Celera assembler and assembly polishing by Quiver [14]. Quality trimming was performed during the preassembly stage of HGAP. This whole genome sequencing project has been deposited at DDBJ/ENA/GenBank under the accession numbers MCFU00000000, MCFV00000000 and MCFW00000000 for isolates #1, #12 and #13, respectively. The genome assemblies described in this paper are versions MCFU01000000, MCFV01000000, and MCFW01000000. Assembled genomes were annotated using the Pathosystems Resource Integration Center (PATRIC) annotation service, and the proteomes were compared with vancomycin-susceptible ATCC *E*. *faecalis* 29212 [15]. Assembled genomes were analyzed using the PubMLST, ResFinder, VirulenceFinder and PATRIC databases to confirm sequence type and identify genes of interest [13, 15–17]. The ATCC 29212 genome used for comparison was retrieved from GenBank under the accession number CP00816 [18]. Identification thresholds were set at 98% identity over a minimum length of 60% for ResFinder, and 95% identity over a length of 60% for VirulenceFinder. DNA gyrase (*gyrA*) and topoisomerase IV subunits A and B (*ParC* and *ParE*) FASTA protein sequences were compared between macaque isolates and *E*. *faecalis* reference strain ATCC 29212 using the multiple sequence alignment tool on PATRIC to identify amino acid polymorphisms contributing to fluoroquinolone resistance.

### Static Biofilm Assay

Biofilm formation was assayed by measuring crystal violet binding, according to previously published protocols with slight modification [19, 20]. Macaque *E*. *faecalis* isolates, and positive control ATCC *E*. *faecalis* 29212, were plated on tryptic soy agar containing 5% sheep blood, and incubated at 37°C in 5% CO_2_ for 24 h. Sterile 1 μl disposable loops were used to inoculate isolates into 5 ml of tryptic soy broth supplemented with 1% glucose (w/v). Following overnight incubation at 37°C, the optical density at 600 nm was recorded using a microtiter plate spectrophotometer (Epoch, Biotek Instruments, Inc., Winooski, VT). For each isolate, 2 μl of overnight culture was diluted into 198 μl of tryptic soy broth supplemented with 1% glucose, in triplicate, in 96 well polystyrene plates. For comparison, 4 negative controls containing glucose-supplemented tryptic soy broth without bacterial inoculum were included. Microtiter plates were incubated at 37°C, shaking at 100 rpm and biofilm formation was evaluated at 24 h. Following aspiration of medium and planktonic cells, wells were washed three times with 200 μl of phosphate-buffered saline, inverted, and air-dried for 45 min. The remaining biofilm was fixed with 200 μl of methanol for 20 min, and plates were inverted and air-dried for an additional 45 min. Biofilm was stained with 150 μl of 1% crystal violet for 20 min. Excess stain was removed via aspiration, followed by rinsing under running tap water. After again air-drying, biofilm-bound crystal violet was solubilized via the addition of 150 μl ethanol for 25 min. The absorbance of the extracted dye was measured at 570 nm using a microtiter plate spectrophotometer, and adjusted for the absorbance of the negative control. Biofilm optical density was normalized to the initial bacterial cell mass by dividing the absorbance of the extracted dye by the OD_600_ of the initial inoculum. Each biofilm test was run in triplicate, and biological replicates were repeated in triplicate on a separate day, independently, to confirm results. Beeswarm plots for each isolate did not qualitatively show systematic bias due to batch effects between experiments, thus data was pooled for analysis (S1 Fig). Beeswarm plots were generated using the Python programming language (Python 3.5.2, matplotlib 1.5.1, seaborn 0.8.0), and Bayesian analysis was done using PyMC3 (ver 3.0 rc2) (S1 Fig). Data was analyzed using a Kruskal-Wallis test with Dunn's multiple correction in GraphPad Prism (GraphPad Software, Inc., La Jolla, CA) with P <0.05 considered significant.

### Biofilm Assessment under Flow

A flow cell system was assembled and sterilized as previously described [21, 22], and biofilm formation was assessed using BM2 medium [62 mM potassium phosphate buffer (pH 7), 7 Mm (NH_4_)_2_SO_4_, 2 mM MgSO_4_, 10 μM FeSO_4_, 0.4% (wt/vol) glucose]. The chambers were inoculated with overnight cultures of *E*. *faecalis* isolates #1, #5, #12 and #13, and bacteria were allowed to attach to the surface of the flow cell chambers for 7 h 40 min. Bacteria were then grown for 43 h under a flow of 2.4 ml/min, after which viable bacteria were stained with SYTO-9 and subsequently visualized using a confocal laser scanning microscope (Zeiss LSM 700). Three-dimensional reconstructions were generated using the Imaris software package (Bitplane AG).

## Results

### Aerobic Culture and Sensitivity

**Fig 2:**
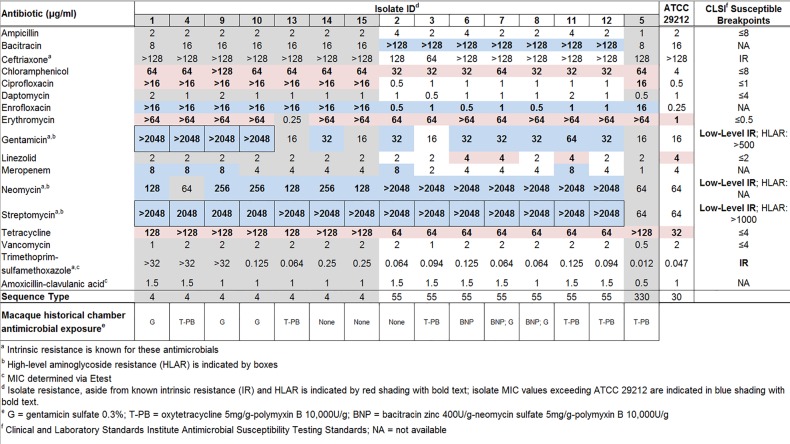
Minimum Inhibitory Concentration (MIC) via Broth Microdilution and Etest Results for Macaque Chamber *E*. *faecalis* Isolates as Compared to ATCC *E*. *faecalis* 29212. Sequence types and historical recording chamber antimicrobial exposure are indicated for each macaque isolate.

From the 45 cephalic recording chambers sampled, 72 aerobic bacterial isolates were examined, with the most common species being *Staphylococcus aureus* (n = 20), *Enterococcus faecalis* (n = 15) and *Proteus mirabilis* (n = 6) (Table 3). The vast majority of cephalic recording chambers grew polymicrobial cultures with a mean and standard deviation of 2.8 ± 1.5 different species (Table 4). Kirby-Bauer testing revealed that while *S*. *aureus* isolates were susceptible to the majority of antimicrobials tested (data not shown), *E*. *faecalis* and *Proteus* spp. isolates were multi-drug resistant (Table 5, S2 Table). Due to the prevalence of *E*. *faecalis* in our colony, we further investigated the antimicrobial resistance characteristics of these isolates. MIC determination for the 15 *E*. *faecalis* isolates confirmed high-level resistance to aminoglycosides, as well as resistance to bacitracin, chloramphenicol, fluoroquinolones, erythromycin, tetracycline, and trimethoprim-sulfamethoxazole (Fig 2). Aminoglycoside resistance was especially prevalent, with 14/15 isolates displaying high-level resistance to streptomycin, 7/15 isolates resistant to neomycin, and 4/15 isolates with high-level resistance to gentamicin. No resistance to vancomycin, linezolid or daptomycin was identified among isolates. Five isolates (ST 4 isolates #1, #4, #9 and ST 55 isolates #2 and #11) displayed an elevated meropenem MIC (8 μg/ml) compared to ATCC 29212 (4 μg/ml). No CSLI susceptibility breakpoint for meropenem has been established for *E*. *faecalis*; however, 8 μg/ml is within the previously reported MIC_90_ [23, 24]. Isolates #6, #7, #11, and ATCC 29212, possessed MICs to linezolid (4 μg/ml) above the CLSI breakpoint of ≤2 μg/ml. Previous literature suggests these isolates fall within the MIC_90_ range for the enterococci, and should be classified as intermediate resistant [25, 26].

**Table 3:** Aerobic Bacterial Culture Results from 45 Chambers^1^of Research Macaques (n = 25).

Bacterial Isolate	Number (%) of Isolates
*Staphylococcus aureus*	20 (27.8)
*Enterococcus faecalis*	15 (20.8)
*Proteus mirabilis*	6 (8.3)
Group C β*-Streptococcus dysgalactiae*	5 (6.9)
*Proteus vulgaris*	4 (5.6)
*Staphylococcus intermedius*	4 (5.6)
*Enterococcus avium*	3 (4.2)
*Escherichia coli*	2 (2.8)
Group A β*-Streptococcus pyogenes*	2 (2.8)
*Leuconostoc* spp.	2 (2.8)
*Streptococcus uberis*	2 (2.8)
*Aerococcus viridans 2*	1 (1.4)
*Enterococcus hirae*	1 (1.4)
Group F β*-Streptococcus constellatus*	1 (1.4)
*Proteus penneri*	1 (1.4)
*Proteus* sp.	1 (1.4)
*Staphylococcus epidermidis*	1 (1.4)
*Staphylococcus xylosus*	1 (1.4)
**Total**	**72 (100)**

^1^Cultures were pooled for animals with multiple recording chambers

**Table 4:** The Majority of Macaque Chambers Display Polymicrobial Colonization.

Number of Isolates	# (Percentage) of Macaques
0	1 (4%)
1	4 (16%)
2	7 (28%)
3	5 (20%)
4	4 (16%)
5	3 (12%)
6	1 (4%)

**Table 5:** Kirby Bauer Disk Diffusion Results for 15 Macaque Chamber *E*. *faecalis* Isolates.

Antibiotic	# Isolates tested	# Sensitive Isolates (%)	# Intermediate Isolates (%)	# Resistant isolates (%)
AMP	15	11 (73%)	0	4 (27%)
AMC	15	11 (73%)	0	4 (27%)
B	15	2 (13%)	5 (33%)	8 (53%)
CR	15	0	0	15 (100%)
E	15	0	1 (7%)	14 (93%)
GM	15	5 (33%)	2 (13%)	8 (53%)
OX	15	0	0	15 (100%)
SXT	15	1 (7%)	0	14 (93%)
ENO	15	0	4 (27%)	11 (73%)
TE	15	0	0	15 (100%)
T	15	0	0	15 (100%)
CRO	15	0	0	15 (100%)
D	5	1 (20%)	3 (60%)	1 (20%)
N	15	0	1 (7%)	14 (93%)
CZ	15	0	1 (7%)	14 (93%)
PB	15	0	0	15 (100%)
VA	13	1 (8%)	5 (38%)	7 (54%)

Sensitive, Intermediate and Resistant designations were determined by measuring the zone of inhibition (mm) and comparing to CLSI guidelines^9^. AMP–Ampicillin, AMC–Amoxicillin/Clavulanic Acid, B–Bacitracin, CR–Cephalothin, E–Erythromycin, GM–Gentamicin, OX–Oxacillin, SXT–Trimethoprim-Sulfamethoxazole, ENO–Enrofloxacin, TE- Tetracycline, T- Oxytetracycline, CRO- Ceftriaxone, D- Doxycycline, N- Neomycin, CZ- Cefazolin, PB- Polymixin B, VA- Vancomycin

### Sequencing and Annotation

Multi-locus sequencing typing identified three sequence types present among the 15 isolates (Fig 2). Most were either ST 4 (n = 7) or ST 55 (n = 7), and a single isolate (isolate #5) was identified as ST 330. Proteome predictions identified 260, 221 and 208 unique proteins encoded by isolates #1 (ST 4), #12 (ST 55) and #13 (ST 4), respectively, not included in those encoded by *E*. *faecalis* ATCC 29212 (ST 30; S3 Table). The vast majority (359/691) of these genes were annotated as hypothetical and most likely reside on mobile elements. Mobile element related genes identified included transposases, type III restriction-modification system proteins, phage proteins, transcriptional regulator proteins, and others, which were more abundant in the ST 4 isolates than ST 55. An additional distinguishing feature between the ST 4 isolates and ST 55 isolate is the presence in the latter of the CRISPR (clustered regularly interspaced short palindromic repeats)-*cas* (CRISPR-associated genes) system. While both ST 4 isolates lacked CRISPR-*cas* genes, a type II-A CRISPR-*cas* system was identified in ST 55 isolate 12, based on the presence of *cas1*, *cas2*, *csn1* and *csn2 [27].*

### Antimicrobial Resistance Genes

A variety of acquired antimicrobial resistance genes were identified in the genomes of isolates #1, #12 and #13 using ResFinder and PATRIC (Table 6). Four genes encoding resistance to aminoglycosides were identified, including *str* in ST 4, *aph(3’)-III* and *ant(6)-Ia* in ST 55, and the bifunctional aminoglycoside-modifying enzyme *aac(6’)-aph(2”)* in ST 4 isolate #1. Other genes noted among the three isolates included macrolide resistance genes *lsa(A)* and *erm(B)*, tetracycline resistance genes, *tet(M)*, *tet(S)*, and *tet(L)*, and the chloramphenicol resistance gene *cat*. Increased trimethoprim-sulfamethoxazole resistance was identified in ST 4 isolate #1 encoded by *dfrG*. The bacitracin resistance genes *bcrA* (ATP binding domain of ATP transporter), *bcrB* (membrane-bound permease of ABC transporter) and *bcrR* (regulatory protein of the *bcrABD* operon) were identified in ST 55 isolate #12 using the specialty gene finder in PATRIC [28]. Multiple sequence alignment of the genes encoding topoisomerase IV subunit A (*parC*) and DNA gyrase subunit A (*gyrA*) revealed single amino acid polymorphisms in *parC* codon 80 (Ser to Ile) in *gyrA* codon 83 (Ser to Ile) in both ST 4 fluoroquinolone-resistant isolates as compared to the less fluoroquinolone-resistant ST 55 isolate #12 and ATCC 29212. No single amino acid polymorphisms were detected in topoisomerase IV subunit B (*parE*).

**Table 6:** Selected Antimicrobial Resistance MIC Results and Resistance Genes Identified from Whole Genome Sequence Data.

Antibiotic (μg/ml)	Isolate Id ^c^				Relevant Resistance Genes Identified^d^
	1	13	12	ATCC 29212	
Bacitracin	8	16	**>128**	16	*bcrA*, *bcrB*, *bcrR* (isolate 12)
Chloramphenicol	**64**	**64**	**32**	4	*cat* (all isolates)
Ciprofloxacin	**>16**	**>16**	1	0.5	*parC* (S80I) and *gyrA* (S83I) mutations (isolates 1 and 13)
Enrofloxacin	**>16**	**>16**	1	0.25	*parC* (S80I) and *gyrA* (S83I) mutations (isolates 1 and 13)
Erythromycin	**>64**	0.25	**>64**	**1**	*ermB* (isolates 1 and 12), *lsa(A)* (all isolates and 29212)
Gentamicin^a^	**>2048**	16	**32**	16	*aac(6’)-aph(2”)* (isolate 1)
Neomycin^a^	**128**	**128**	**>2048**	64	*aph(3’)-III* (isolate 12)
Streptomycin^a^	**>2048**	**>2048**	**>2048**	64	*str* (isolates 1 and 13), *aph(3’)-III* and *ant(6)-Ia* (isolate 12)
Tetracycline	**128**	**128**	**64**	**32**	*tetM* (all isolates and 29212), *tetS* (isolates 1 and 13), *tetL* (isolate 12)
Trimethoprim-sulfamethoxazole^b^	**>32**	0.064	0.094	0.047	*dfrG* (isolate 1)
**Sequence Type**	4	4	55	30	

^a^ High-level aminoglycoside resistance (HLAR) is indicated by boxes

^b ^MIC determined by Etest

^c ^Isolate resistance, aside from known intrinsic resistance and HLAR, is indicated by red shading with bold text; isolate MIC values exceeding ATCC 29212 are indicated in blue shading with bold text.

^d ^Assembled genomes were uploaded to ResFinder with a 98% threshold for gene identification and a minimum length of 60%. The PATRIC specialty gene finder tool was used to identify *bcrABR* genes. Multiple sequence alignment was performed in PATRIC to identify amino acid polymorphisms conferring fluoroquinolone resistance.

### Virulence Factor and Biofilm Formation-Associated Genes

Genes encoding virulence factors from isolates #1, #12 and #13 were identified using VirulenceFinder and the specialty feature tool in PATRIC (Table 7). Genes associated with the cytolysin toxin (*cylA*, *cylB*, *cylL and cylM*) were identified in both ST 4 isolates. Many genes associated with biofilm formation were identified, including aggregation substance (*agg*), enterococcal surface protein (*espfs*), endocarditis and biofilm-associated pili genes (*ebpA*, *ebpB*, *ebpC*), collagen adhesion precursor (*ace*), gelatinase toxin (*gelE*) and sortase (*SrtA*). The ST 4 isolates possessed more biofilm-associated factors compared to ST 55 isolate 12, which lacked aggregation substance and gelatinase. Because of this finding, we further hypothesized that ST 4 isolates would produce more biofilm than ST 55 isolates and performed biofilm assays to evaluate this hypothesis. Other virulence factors identified included sex pheromone-associated genes (*cad*, *cCF10*, *camE*, *cOB1*), the cell wall adhesion expressed in serum gene (*efaAfs*), the enterococcal Rgg-like regulator gene associated with macrophage persistence (*ElrA*), hyaluronidases (*hylA*, *hylB*), and the thiol peroxidase gene to protect against oxidative stress (*tpx*) (Table 7)[29–36]

**Table 7:** Acquired Virulence Factor Genes Identified Using VirulenceFinder^a^ and PATRIC^b^.

		ST 4		ST 55	ST 30
Virulence Factor Function^c^	Gene	Isolate 1	Isolate 13	Isolate 12	ATCC 29212^d^
**Collagen adhesin precursor**	***ace***	**X**	**X**	**X**	**X**
**Aggregation substance**	***agg***	**X**	**X**		**X**
**Endocarditis and biofilm-associated pili genes**	***ebpA***	**X**	**X**	**X**	**X**
	***ebpB***	**X**	**X**	**X**	**X**
	***ebpC***	**X**	**X**	**X**	**X**
**Cell wall adhesin expressed in serum**	***efaAfs***	**X**	**X**	**X**	**X**
**Enterococcal surface protein**	***esp***	**X**	**X**	**X**	
**Gelatinase toxin (metalloendoprotease)**	***gelE***	**X**	**X**		**X**
**Sortase A**	***SrtA***	**X**	**X**	**X**	**X**
**Cytolysin (hemolysin-bacteriocin)**	***cylL***	**X**	**X**		**X**
**Post-translational cytolysin modification**	***cylM***	**X**	**X**		**X**
**Transport of cytolysin**	***cylB***	**X**	**X**		**X**
**Activation of cytolysin**	***cylA***	**X**	**X**		**X**
Sex pheremone	*cad*	X	X	X	X
Sex pheremone cAM373 precursor	*camE*	X	X	X	X
Sex pheremone	*cCF10*	X	X	X	X
Sex pheremone	*cOB1*	X	X	X	X
Enterococcal Rgg-like regulator	*ElrA*	X	X	X	X
Hyaluronidase	*hylA*	X	X	X	X
	*hylB*			X	
Thiol peroxidase (oxidative stress resistance)	*tpx*	X	X	X	X

^a^ Assembled genomes were uploaded to ResFinder with a 95% threshold for gene identification and a minimum length of 60%. https://cge.cbs.dtu.dk/services/VirulenceFinder/

^b^ The PATRIC Specialty Gene Finder tool was used to confirm virulence factors following annotation.

^c^ Genes associated with biofilm production are designated with blue shading and bolded text and genes associated with cytolysin toxin production are designated with red shading and bolded text.

^d^ Genome was obtained from GenBank accession CP008816.

### Biofilm Production

**Fig 3:**
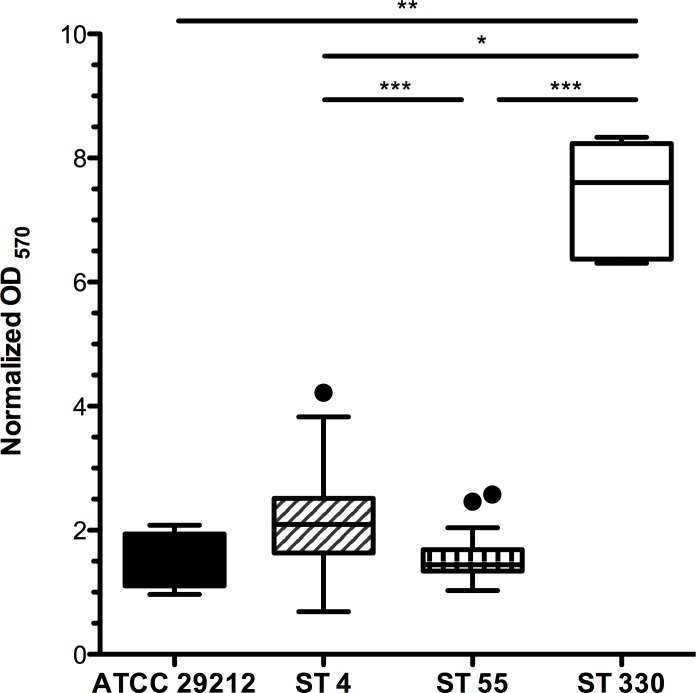
24 hour Biofilm Production for 15 *E*. *faecalis* Isolates Assessed by Crystal Violet Staining. ST 4 and ST 330 isolates produce significantly more biofilm than ST 55 isolates (see also S4 Table). Mean normalized OD_570_ for pooled data were 2.016 ±.0.016 for ST 4 isolates, 1.500 ± 0.2942 for ST 55 isolates, 8.191 ± 0.1489 for the ST 330 isolate and 1.894 ± 0.1833 for the ATCC 29212 control strain.

**Fig 4:**
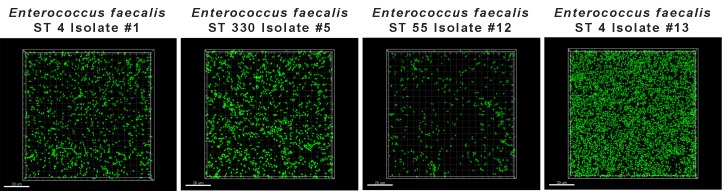
Biofilm Growth of *E*. *faecalis* in Flow Cell Chambers. Attached cells grown in flow cell chambers for 43 h were stained green with SYTO-9, and visualized at 63X magnification. Images are representative for each isolate.

Significant differences in biofilm production, as measured by crystal violet binding, were noted among the *E*. *faecalis* isolates (Fig 3, P < 0.0001, Kruskal-Wallis with Dunn’s Multiple Comparison). The ST 330 isolate was a robust biofilm former, producing more biofilm than the ST 4, ST 55 isolates, or control ATCC *E*. *faecalis* 29212 (Fig 3, S4 Table). Flow cell assays also revealed that ST 55 isolate #12 exhibited a biofilm-deficient phenotype when compared to ST 4 isolates #1, and #13 and ST 330 isolate #5 (Fig 4). On the other hand, ST 4 isolate #13 showed a hyper-biofilm phenotype compared to all other isolates tested (Fig 4).

## Discussion

*Enterococcus faecalis* is an important cause of healthcare-associated infections, and is the third most common cause of central line-associated bloodstream infections, the fifth most common cause of catheter-associated urinary tract infections, and the sixth most common cause of healthcare-associated infections overall [37]. The enterococci possess mechanisms of both acquired and intrinsic resistance to multiple antimicrobial agents, making nosocomial infection especially difficult to treat successfully. In this study, our *E*. *faecalis* isolates were identified from chronic cephalic implants, and are likely associated with biofilm. Most isolates belonged to two multi-drug resistant sequence types and we identified a variety of resistance genes and virulence genes from representative sequenced whole genomes. Minimum inhibitory concentration testing via broth dilution was elected to both confirm initial Kirby Bauer disk diffusion results and quantify the magnitude of resistance. There were some discrepancies between disk diffusion and MIC results; specifically, disk diffusion suggested some isolates displayed resistance to ampicillin, amoxicillin-clavulanic acid and vancomycin. MIC testing found all isolates to be below the resistant breakpoint for ampicillin (8 μg/ml) and vancomycin (4 μg/ml). While no CLSI resistance breakpoint is noted for amoxicillin-clavulanic acid, all isolates and the ATCC control strain 29212 had an MIC between 0.5–1.5 μg/ml. Discrepancies between disk diffusion classification and MIC susceptibilities may be attributed to not recording disk diffusion results at exactly 24 hours, or error in agar inhibitory zone measurement.

Antimicrobial resistance profiles obtained by MIC and Etest related well to antimicrobial resistance genes identified from representative whole genome sequence data (Table 6). Gentamicin MIC profiles also matched well, but not perfectly, with a history of gentamicin exposure. Specifically, *E*. *faecalis* isolates #1, #7, #8, #9 and #10 (5/15 isolates; 33%) were isolated from macaques 1, 16, 18 and 19 with a history of gentamicin sulfate administration into their recording chambers. While ST 4 isolates #1, #9 and #10 display high-level gentamicin resistance (HLGR), ST 55 isolates 7 and 8 from macaque 16 lacked HLGR. The *aac(6’)-aph(2”)* gene has previously been established to confer HLGR, which is supported by the differences in MIC between HLGR ST 4 isolate #1 (>2048 μg/ml) and non-HLGR isolate #13 (16 μg/ml) [38]. The presence of the *aac(6’)-aph(2”)* gene was confirmed to be in close proximity to genes encoding plasmid recombination enzymes in ST 4 isolate #1. The acquisition of such mobile-element-derived genes may be inhibited by an intact CRISPR-*cas* system, which was identified in ST 55 isolate #12 [39]. Additionally, both ST 4 and ST 55 isolates display high-level streptomycin resistance, and ST 55 isolates also demonstrate increased resistance to neomycin. The presence of high-level aminoglycoside resistance is significant because it abolishes synergistic treatment with the combination of an aminoglycoside and cell-wall inhibitor [38, 40].

The plasmid-derived *bcrABD* operon with an upstream regulator *bcrR* has previously been identified to confer bacitracin resistance in *E. faecalis [28]*. We identified *bcrA*, *bcrB* and *bcrR* in ST 55 isolate #12 with 89%, 89% and 94% homology, respectively, to previously reported bacitracin resistance genes [28]. Previous mutagenesis experiments have established that the *bcrD* gene encoding undecaprenol kinase is not required for high-level bacitracin resistance [28]. While macaque 8 (ST 55 isolate #12) does not have a history of bacitracin exposure, macaques 15 and 16 (ST 55 isolates 6, 7 and 8) did have chamber exposure to triple-antibiotic ointment containing bacitracin and are our hypothesized source of selective pressure in our colony.

Multiple genes conferring resistance to tetracyclines were identified, including both ribosomal protection genes and genes encoding efflux pumps [41]. Macaques 5, 6, 8 and 9, from which *E*. *faecalis* isolates #3, #4, #5, #11, #12 and #13 were identified, had a history of exposure to combination oxytetracycline-polymyxin B ointment inside recording chambers (Fig 2, S1 Table). Isolates #4, #5 and #13 displayed a higher MIC (≥ 128 μg/ml) than isolates #3, #11 and #12, suggesting that the *tetS* gene may be responsible for conferring increased tetracycline resistance as compared to *tetL* and *tetM*.

Two single amino acid polymorphisms previously identified to confer fluoroquinolone resistance were observed in genes encoding DNA gyrase subunit A (*gyrA*) and topoisomerase IV subunit A (*parC*) in both ST 4 isolates sequenced [42, 43]. Enrofloxacin is the most commonly used fluoroquinolone in our colony and has been administered systemically both perioperatively, and therapeutically for clinical cases of wounds or suspected meningitis. Macaque 3 had chamber exposure to dilute enrofloxacin but *E*. *faecalis* was not isolated from the chamber of this individual.

The ability of the enterococci to form biofilms contributes to the pathogenicity of implant-associated infections, as mature biofilms allow *E*. *faecalis* to withstand antimicrobial agents at 10-1000-fold greater concentrations than those required to control planktonic bacteria [44]. We identified virulence factor genes associated with biofilm formation including aggregation substance (*agg*), enterococcal surface protein (*esp*), adhesion of collagen from *E*. *faecalis* (*ace*), gelatinase (*gelE*), endocarditis and biofilm-associated pili genes (*Ebp*) and sortase A (S*rtA*) [35, 45–48]. ST 4 isolates produced significantly more biofilm than ST 55 isolates. Interestingly, one ST 330 isolate formed a robust static biofilm, and additional genetic analysis will be needed to relate this phenotype to genotype (Fig 3). Whole genome sequencing of representative strains showed that ST 55 isolate 12 lacked both *agg* and *gelE*, which were present in ST 4 isolates. Nevertheless, we cannot attribute increased biofilm production by ST 4 to the presence of *agg* and *gelE*, as the *agg*- and *gelE*-positive ATCC 29212 *E*. *faecalis* strain showed no significant differences in biofilm-forming ability compared to either ST 4 or ST 55 isolates. It is probable that increased biofilm production by ST 4 and ST 330 isolates, compared to ST 55 isolates, is polygenic in nature, as multiple genes including *esp*, *agg*, *gelE*, and *srtA* have been individually shown to contribute to biofilm formation [49–52]. As well as genes associated with biofilm formation, we identified the presence of genes encoding the cytolysin toxin in both ST 4 isolates sequenced. The cytolysin toxin has been shown to increase the lethality of infection in multiple species, including mice, rabbits and humans, and can act synergistically with aggregation substance to increase lethality of infection in a rabbit endocarditis model [53, 54]. Due to the rarity of confirmed *Enterococcus*-associated implant complications we cannot definitively assess how ST 4 isolates bearing the cytolysin toxin contribute to pathogenicity in macaques with chronic implants.

Our macaque colony models an environment with many similarities to humans possessing indwelling devices in a long-term care facility. Macaques are housed in a high-density environment, with approximately 12 animals per housing room. Human intensive care units (ICUs) vary in size, but recent reports have suggested a median ICU bed density in the range of 12–30 beds [55, 56]. Similar to a mixed-population ICU, macaques are housed in a mixed population, and surgically naïve individuals and individuals with cephalic implants of varying duration can be pair-housed or housed in close proximity within the same room. Besides topical antimicrobial exposure inside recording chambers, macaques in our vivarium are exposed to systemic antimicrobial therapy during the perioperative period and when clinically indicated, such as prophylactic treatment for wounds resulting from an altercation with a conspecific. This chronic intermittent antimicrobial exposure provides a selective pressure for resistant isolates to emerge, as well as provide a niche for intestinal overgrowth and permit breakdown of colonization resistance [57]. MIC testing suggests that our *E*. *faecalis* isolates remain susceptible to ampicillin, amoxicillin-clavulanic acid, vancomycin, daptomycin and meropenem. Vancomycin, daptomycin and meropenem are not used in our facility and will continue to be excluded from clinical use while isolates remain susceptible to second-generation penicillins, such as ampicillin.

Finally, chronic cephalic implants serve as a nidus for biofilm formation and persistent infection. A variety of bacterial species, including *Staphylococcus*, *Enterococcus*, and *Corynebacterium* spp. have been previously cultured from the skin margins of macaque cephalic implants [4, 5]. It is unknown whether chamber colonization results from translocation of skin flora or from fecal contamination. Because of the parallels between the macaque colony under study and a human hospital environment, it is not surprising to see similarities in identified pathogens. Specifically, the main two *E*. *faecalis* sequence types, ST 4 and ST 55, detected in the macaque population have been previously identified as agents of human infection [58–61].While the majority of chamber infections are subclinical in the macaque colony, the potential importance of meningitis, cephalic abscess, and bacteremia/septicemia to animal health maintenance necessitates a thorough understanding of the bacterial species colonizing implants, their antimicrobial resistance profiles and the appropriate use of antimicrobials, when warranted. The samples analyzed here were recovered from aerobic cultures, and so it remains possible, perhaps likely, that additional anaerobic microbes are present in the mixed microbial biofilms, which remain to be identified.

With improved understanding of multi-drug resistant bacteria colonizing research macaques, veterinary protocols have been updated to address judicious use of antimicrobials in our facility. Topical instillation of antimicrobial agents by investigators is now prohibited and only allowed under veterinary supervision. The veterinary staff directs regular disinfection of cephalic recording chambers, with frequency of disinfection dependent on culture results, as well as the amount and appearance of discharge. Disinfection protocols for cephalic recording chambers have been standardized to 1–2% povodone iodine solution and saline under strict aseptic technique. Perioperative trimethoprim-sulfamethoxazole and enrofloxacin have been replaced by cefazolin, and post-operative ceftriaxone use is restricted to surgical procedures involving a craniotomy. Animals with suspected meningitis undergo thorough diagnostic work-up, including culture and sensitivity testing of isolates in cerebrospinal fluid to guide choices in antimicrobial therapy and minimize further spread of bacterial resistance genes within the macaque colony.

## Conclusions

Chronic cephalic implants in macaques used for cognitive neuroscience research can serve as a nidus of multi-drug resistant bacterial pathogens. We have identified polymicrobial colonization as a common feature, and noted that *S*. *aureus*, *Proteus spp*. and *E*. *faecalis* are the most prevalent organisms associated with these implants. Multi-locus sequence typing revealed that two major *E*. *faecalis* sequence types associated with human infection are present in our colony. Cephalic implants also serve as a minimally invasive access location for studying biofilm formation on medical-grade biomaterials to provide better insight on the mechanisms of enterococcal persistence in a healthcare setting. Further investigation is needed to understand how *S*. *aureus*, *Proteus* and other bacteria contribute to chronic polymicrobial chamber infections. Future studies will investigate *E*. *faecalis* colonization of implanted devices, feces and other sites at multiple time points to evaluate sources and reservoirs for these hospital pathogens. Additional investigation using *in vitro* biofilm models will help elucidate the roles of varying combinations of pathogens and the effectiveness of different disinfection and treatment regimens in controlling implant colonization.

## Supporting Information

S1 Fig Beeswarm Plot of Crystal Violet Biofilm Experimental Data. (TIF)

S1 Table Cleaning Frequency and Cephalic Recording Chamber Maintenance Survey. (XLSX)

S2 Table Kirby Bauer Antimicrobial Disk Diffusion Results for 12 Proteus spp Macaque Chamber Isolates. (XLSX)

S3 Table Unique Proteins from E. faecalis Isolates #1, #12 and #13 as Compared to ATCC 29212. (XLSX)

S4 Table Crystal Violet Biofilm Assay Optical Density Data. (XLSX)
