# Supplementary figures and images for: Characterization of Multi-Drug Resistant Enterococcus faecalis Isolated from Cephalic Recording Chambers in Research Macaques (Macaca spp.)
Source: PLoS One. 2017 Jan 12;12(1):e0169293. doi: 10.1371/journal.pone.0169293 (PMC5231353; doi:10.1371/journal.pone.0169293)

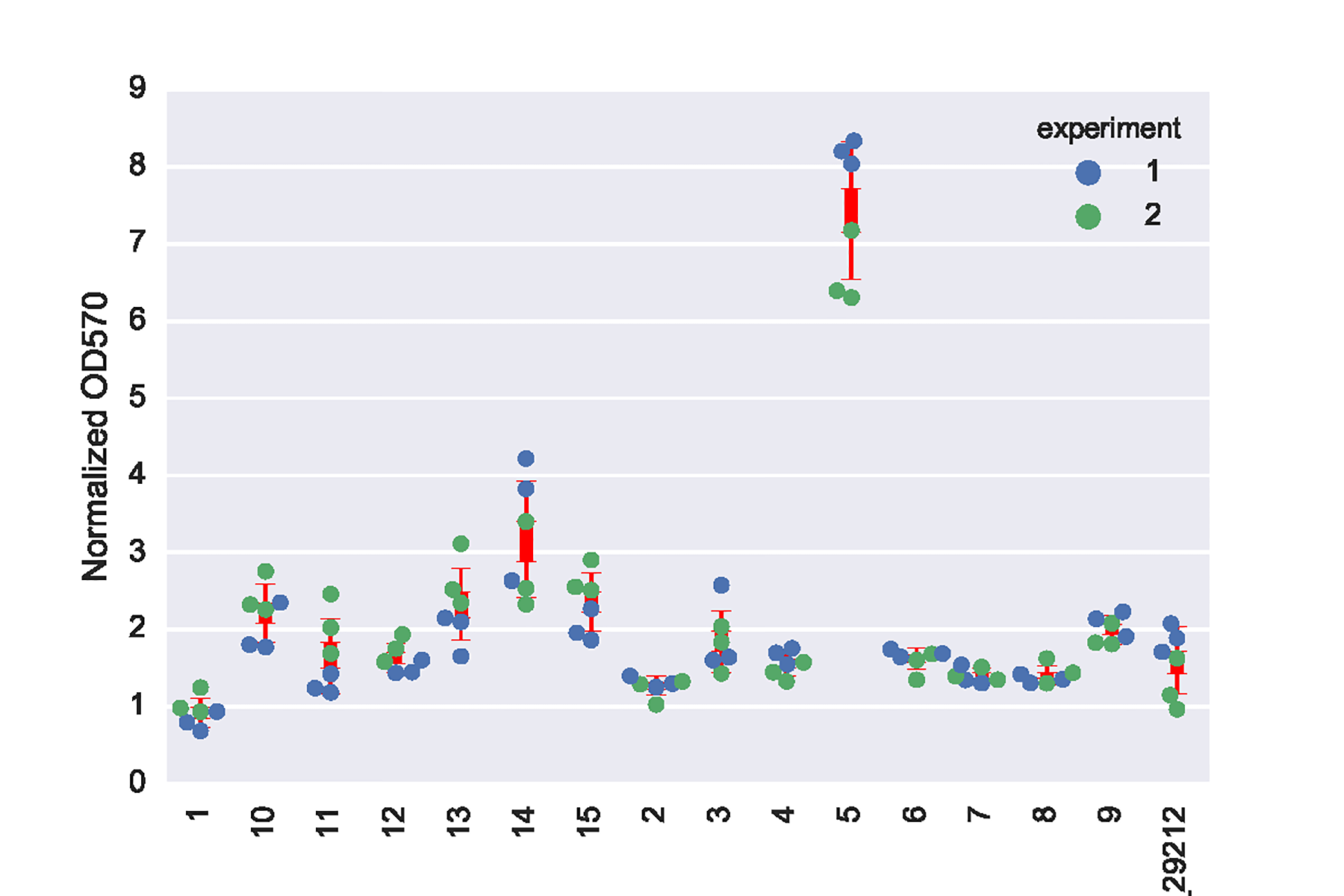

Supplement: S1 Fig — (TIF) [file pone.0169293.s001.tif]
